# Dual-Strategy Design Based on Polymer–Matrix Composite Cathode and Coated Separator for High-Performance Lithium–Iron Disulfide Batteries

**DOI:** 10.3390/ma18174058

**Published:** 2025-08-29

**Authors:** Fan Zhang, Qiang Lu, Jiachen Li, Qiongyue Zhang, Haotian Yu, Yahao Wang, Jinrui Li, Haodong Ren, Huirong Liang, Fei Shen, Xiaogang Han

**Affiliations:** 1 School of Electric Power, Civil Engineering and Architecture, Shanxi University, Taiyuan 030000, China; fanzhang@sxu.edu.cn (F.Z.);; 2 State Key Laboratory of Electrical Insulation and Power Equipment, School of Electrical Engineering, Xi’an Jiaotong University, Xi’an 710049, China; 3 Shanxi Yudean Energy Company Limited, Taiyuan 030006, China

**Keywords:** lithium–iron disulfide battery, polymer-based composite cathode, coated separator, polysulfide shuttle effect, cycle stability

## Abstract

Lithium–iron disulfide (Li-FeS_2_) batteries are plagued by the polysulfide shuttle effect and cathode structural degradation, which significantly hinder their practical application. This study proposes a dual-strategy design that combines a polyacrylonitrile–carbon nanotube (PAN-CNT) composite cathode and a polyvinylidene fluoride (PVDF)-conductive carbon-coated separator to synergistically address these bottlenecks. The PAN-CNT binder establishes chemical anchoring between polyacrylonitrile and FeS_2_, enhancing electronic conductivity and mitigating volume expansion. Specifically, the binder boosts the initial discharge capacity by 35% while alleviating the stress-induced pulverization associated with volume changes. Meanwhile, the PVDF-conductive carbon-coated separator enables effective polysulfide trapping via dipole–dipole interactions between PVDF’s polar C-F groups and Li_2_S_x_ species while maintaining unobstructed ion transport with an ionic conductivity of 1.23 × 10^−^^3^ S cm^−^^1^, achieving a Coulombic efficiency of 99.2%. The electrochemical results demonstrate that the dual-modified battery delivers a high initial discharge capacity of 650 mAh g^−1^ at 0.5 C, with a capacity retention rate of 61.5% after 120 cycles, significantly outperforming the control group’s 47.5% retention rate. Scanning electron microscopy and electrochemical impedance spectroscopy confirm that this synergistic design suppresses polysulfide migration and enhances interfacial stability, reducing the charge transfer resistance from 26 Ω to 11 Ω. By integrating polymer-based functional materials, this work presents a scalable and cost-effective approach for developing high-energy-density Li-FeS_2_ batteries, providing a practical pathway to overcome key challenges in their commercialization.

## 1. Introduction

In the pursuit of sustainable energy, high-performance energy storage technologies have emerged as pivotal enablers for modern societal development. Lithium-ion batteries, widely recognized as the cornerstone of modern energy storage systems, underpin a diverse array of applications ranging from portable electronics and electric vehicles to large-scale smart grids [1,2,3]. However, the escalating demands for higher energy density, extended cycle longevity, and reduced cost profiles have catalyzed intensive exploration of alternative battery chemistries. Among these, lithium–iron disulfide (Li-FeS_2_) batteries have garnered significant research interest owing to their exceptional theoretical capacity (894 mAh g^−^^1^) and the abundant, earth-rich nature of iron and sulfur resources [4,5,6].

The exploration of Li-FeS_2_ battery chemistry traces back to the 1980s, with Abraham et al. pioneering their development as primary batteries in 1984. Their work first demonstrated the high theoretical capacity of 894 mAh g^−^^1^, derived from the direct redox reaction between FeS_2_ and lithium metal [4]. Despite this early promise, the advancement of rechargeable (secondary) Li-FeS_2_ batteries has long been impeded by critical challenges. Studies in the 1990s elucidated that FeS_2_ undergoes severe structural degradation during cycling, primarily driven by polysulfide dissolution (e.g., Li_2_S_4_/Li_2_S_2_) and substantial volume expansion (up to 160%), which collectively result in a Coulombic efficiency below 80% [5]. Since the 2000s, strategies such as nanostructuring and carbon composite engineering (e.g., FeS_2_/C nanoparticles) have partially mitigated cycle instability; however, single-component cathode modifications still struggle to concurrently address polysulfide shuttling and interfacial impedance issues. In contrast to the mature commercialization of lithium-ion battery systems (e.g., LiCoO_2_/graphite), research on secondary Li-FeS_2_ batteries remains largely confined to laboratory-scale investigations, with the core challenge persisting in balancing high-capacity delivery and long-term cycling stability [6]. Notably, when paired with a lithium metal anode, Li-FeS_2_ batteries can achieve an energy density of 2600 Wh kg^−^^1^, which far surpasses the 400–600 Wh kg^−^^1^ of traditional lithium-ion batteries [7]. This superior energy density renders them highly promising for advanced applications in electric vehicles, smart grids, and aerospace sectors [8,9,10].

However, Li-FeS_2_ batteries face critical hurdles. A primary issue is the polysulfide shuttle effect [11,12,13]: during charge–discharge, intermediate lithium polysulfides (Li_2_S_x_, 4 ≤ x ≤ 8) form, dissolve in electrolytes, migrate to the anode, react with lithium, and cause irreversible capacity loss, reduced Coulombic efficiency, and shortened cycle life [14,15,16]. This also risks dendrite formation and thermal runaway [17,18,19]. Another challenge is cathode structural degradation [20,21,22]. FeS_2_ undergoes up to 160% volume expansion during lithiation, leading to electrode pulverization, lost electrical contact, and performance decay [23,24,25]. These combined issues limit practical use, demanding effective solutions. 

In recent years, researchers have implemented numerous strategies to address these challenges. For mitigating the polysulfide shuttle effect, approaches including nanostructured FeS_2_ particles [26,27,28] and engineered polysulfide-trapping structures [29,30,31] have been explored. For example, graphene-wrapped FeS_2_ cathodes have been reported to improve capacity retention to 68% over 50 cycles by enhancing electronic conductivity and forming a physical barrier against polysulfide diffusion [32]. However, excessive addition of carbon-based materials in such nanostructured composites usually leads to reduced energy density, as high contents of inactive carbon dilute the overall energy storage capacity [16,33,34,35].

Separator modification is also key. Metal–organic framework (MOF)-coated separators improved Li-S retention to 82% [36] but face scaling issues due to complex synthesis and high cost [37,38,39]. Polymer-based separators, especially those with functional coatings, have attracted increasing attention. For instance, polyvinylidene fluoride (PVDF)-based separators have been modified with different additives to enhance their polysulfide adsorption capabilities [40,41,42]. However, performance enhancements remain limited, and more effective and practical solutions are still urgently needed. Traditional binders such as PVDF struggle to maintain cathode structural integrity during cycling [43,44,45]. New binders with better adhesion and mechanical properties are being explored. Polyacrylonitrile (PAN) has exhibited potential as a binder in lithium–sulfur batteries due to its ability to form strong chemical bonds with sulfur-containing active materials [17,46,47,48]. However, its application in Li-FeS_2_ batteries requires further exploration [49,50,51].

To address these challenges, this study proposes a synergistic dual strategy combining polymer-based functional materials. The polyacrylonitrile–carbon nanotube (PAN-CNT) composite cathode utilizes chemical bonding between nitrile groups of PAN and FeS_2_ to strengthen the interface between the active material and the binder, while the three-dimensional conductive network formed by CNTs improves electron transport capability and mechanical toughness to resist volume expansion. Meanwhile, the PVDF-conductive carbon-coated separator forms a hydrophilic porous layer through a scalable doctor-blade coating process, physically trapping polysulfides through dipole–dipole interactions while maintaining unimpeded lithium-ion transport. This design combines chemical anchoring at the cathode interface with physical adsorption at the separator, achieving dual inhibition of the shuttle effect and structural degradation. By optimizing electrode structure and separator functionality, this study provides a feasible approach for developing high-performance Li-FeS_2_ batteries, helping to overcome key obstacles in their commercialization.

## 2. Materials and Methods

### 2.1. Cathode Fabrication

**Figure 1:**
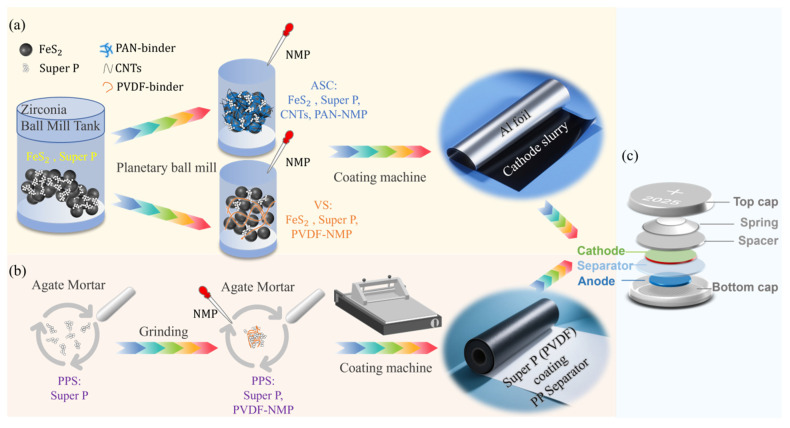
(**a**) Preparation steps for the experimental ASC cathode and control VS cathode. (**b**) Preparation process for the experimental PPS separator. (**c**) Schematic diagram of the full-cell assembly stacking sequence.

Composite cathodes were prepared via a solvent-based slurry coating method. In the experimental group, FeS_2_ nanoparticles (90 wt.%,< 50 nm, Guangdong Yunfu Warner New Energy Co., Ltd.), PAN (molecular weight 85,000, Aladdin), and a mixture of Super P conductive carbon and CNTs (mass ratio 1:1, Super P is analytical grade, Aladdin; CNT purity > 95%, diameter 10–20 nm, Timesnano) were used as the active material, binder, and conductive agent, respectively, with an overall mass ratio of 8:1:1. As shown in Figure 1a, the specific steps were as follows: First, 8 g of commercial FeS_2_ was mixed with 0.5 g of Super P, sealed in a zirconia ball mill jar, and dry-milled for 2 h using a planetary ball mill. Subsequently, 12.5 g of CNT slurry (total solid content = 4 wt.%) and 24 mL of PAN-NMP binder (prepared by adding 0.6 g of PAN to 24 mL of NMP and stirring for 12 h; total solid content = 2.5 wt.%) were added, and the mixture was sealed in a ball mill jar and ball-milled for 12 h. The prepared slurry was coated onto aluminum foil using a 100 μm doctor blade. After coating, the carbon-coated aluminum foil was transferred to a blast drying oven at 60 °C for 2 h, then vacuum-dried at 80 °C for 12 h. The prepared cathode was cut into circular discs with a diameter of 10 mm, denoted as the ASC cathode.

In the control group, the binder was replaced with commonly used PVDF (molecular weight 600,000, Arkema), and the conductive agent was solely Super P conductive carbon (analytical grade, Aladdin), with all other parameters remaining unchanged. The specific steps are shown in Figure 1a, and the control cathode is denoted as the VS cathode.

### 2.2. Separator Modification

Coated separators were prepared using a scalable doctor-blade coating process. In the experimental group, as shown in Figure 1b, carbon-containing binder-coated separators were prepared; specifically, 30 mg of Super P was ground in an agate mortar for 15 min, followed by the addition of 1 mL of binder (PVDF dissolved in NMP, total solid content = 2.5 wt.%) and further grinding for 15 min. The mixed slurry was coated onto the cathode-facing surface of a commercial PP separator (Celgard 2400, 16 μm) using a 100 μm doctor blade without additional pressure. The coated separator was then dried in a blast drying oven at 60 °C for 2 h, cut into circular discs with a diameter of 16 mm, and stored in a glovebox for later use. The experimental separator is denoted as PPS.

The control group used an untreated commercial PP separator, denoted as PP.

### 2.3. Full-Cell Assembly

CR2025 coin cells were assembled in an argon-filled glovebox (H_2_O < 0.1 ppm, O_2_ < 0.1 ppm; SG2400-750TS-H, Wige Gas Purification). The anode was lithium metal foil (diameter 12 mm, thickness 0.5 mm, Tianjin Zhongneng Lithium Industry). The cathode was either the ASC modified composite electrode or the control VS electrode (diameter 10 mm, active material loading 2.0 mg cm^−2^). The separator was either the PPS-coated PP separator or the unmodified PP separator (diameter 16 mm). The electrolyte was 75 μL of 1 M (M: mol/L) lithium bis(trifluoromethanesulfonyl)imide (LiTFSI, 99.9%, Aladdin) in 1,3-dioxolane (DOL)/1,2-dimethoxyethane (DME) (1:1 *v*/*v*). Cells were sealed with a tablet press (JK-FKJ-20, Shenzhen Jingkenuoer, Shenzhen, China) according to the stacking sequence shown in Figure 1c and aged at room temperature for 12 h before testing.

### 2.4. Electrochemical Testing

#### 2.4.1. Cyclic Charge–Discharge Testing

Galvanostatic charge–discharge cycling was performed on a Neware BTS-4000 system (Shenzhen) within a voltage window of 1.0–3.0 V vs. Li^+^/Li at a current density of 0.5 C (1 C = 894 mA g^−1^). Each cycle included a 10 min rest period in both fully charged and discharged states. The capacity retention rate was defined as the ratio of discharge capacity in the nth cycle to the initial capacity, with values presented as averages of three parallel samples to ensure reproducibility. Rate performance tests were conducted at varying current densities (0.1 C, 0.2 C, 0.5 C, 1 C, and back to 0.1 C) over 5 cycles per density.

#### 2.4.2. Electrochemical Impedance Spectroscopy (EIS)

EIS measurements were performed using a Bio-Logic SP-200 workstation (Bio-Logic, Cecile Parise, France) over a frequency range of 1 MHz to 0.1 Hz with a 10 mV AC perturbation amplitude. The charge transfer resistance (Rct) was extracted using the ZView 3.1 software using an equivalent circuit model (Rs(CPE-Rct)), where Rs is the solution resistance and CPE is the constant phase element.

Lithium ionic conductivity was calculated using the alternating current impedance method with the formula σ = L/(R × S), where *σ* is the ionic conductivity (S cm^−1^), *L* is the electrolyte thickness (taken as the separator thickness due to the water absorption of polyolefin separators, ~40 μm), *R* is the ohmic resistance (Ω), and *S* is the electrolyte/electrode interfacial contact area (approximately 2.01 cm^2^, equivalent to the separator area).

#### 2.4.3. Cyclic Voltammetry (CV)

Cyclic voltammetry (CV) tests were performed in a three-electrode configuration (lithium metal counter/reference electrode, FeS_2_ working electrode, stainless steel counter electrode) at a scan rate of 0.1 mV s^−1^ between 1.0 and 3.0 V. The peak current ratio (Ipa/Ipc) and peak separation (ΔEp) were analyzed to assess reaction reversibility and degree of polarization.

#### 2.4.4. Electronic Conductivity Measurement

The electronic conductivity of the cathode films was measured using a DC four-probe tester (RTS-5, Shidai Zhifeng, Beijing, China) at room temperature. The cathode films were cut into rectangular strips (10 mm × 40 mm) and pressed onto a clean glass slide. Four probes were uniformly placed on the film surface with a spacing of 5 mm, and a constant current was applied to measure the voltage drop. The conductivity (σ) was calculated using the formula σ = L/(R × S), where L is the distance between the inner two probes (cm), R is the measured resistance (Ω), and S is the cross-sectional area of the film (cm^2^, calculated as thickness × width).

### 2.5. Microstructural Characterization

#### 2.5.1. Scanning Electron Microscopy (SEM) and Energy-Dispersive X-Ray Spectroscopy (EDS) Analysis

The surface and cross-sectional morphologies of electrodes and separators were observed using a Hitachi SU8010 SEM (5 kV acceleration voltage, Hitachi, Tokyo, Japan). Post-cycling electrodes were disassembled under an argon atmosphere, rinsed with dimethyl ether (DME) to remove electrolyte residues, and sputter-coated with gold (10 nm) before imaging to analyze structural stability and crack density. Elemental distribution in post-cycling separators and electrode samples was characterized using an EDS detector integrated with SEM. EDS surface scanning was performed at 20,000× magnification with a dwell time of 50 ms pixel^−1^.

#### 2.5.2. Brunauer–Emmett–Teller (BET) Surface Area and Pore Size Analysis

The specific surface area and average pore size of the cathodes were determined using a BET surface area analyzer (TriStar II 3020, Micromeritics, Norcross, GA, USA). Prior to testing, samples were degassed at 120 °C under vacuum for 6 h to remove adsorbed moisture and impurities. The specific surface area was calculated using the BET method, while the average pore size was derived using the Barrett–Joyner–Halenda (BJH) model based on the measured structural parameters.

### 2.6. Mechanical Testing

The strength of adhesion of the cathode films to the aluminum current collector was measured using a 180° peel test on a universal testing machine (CMT6104, MTS, Shenzhen, China). Cathode films were cut into strips (10 mm × 50 mm) and manually peeled from the aluminum foil at an initial length of 20 mm. The test was conducted at a peeling speed of 300 mm·min^−1^ and a peeling angle of 180°, with the average peel force recorded over a 20 mm peeling distance. Each sample was tested in triplicate to ensure reproducibility.

## 3. Results and Discussion

### 3.1. Synergistic Enhancement of Electrochemical Performance

**Figure 2:**
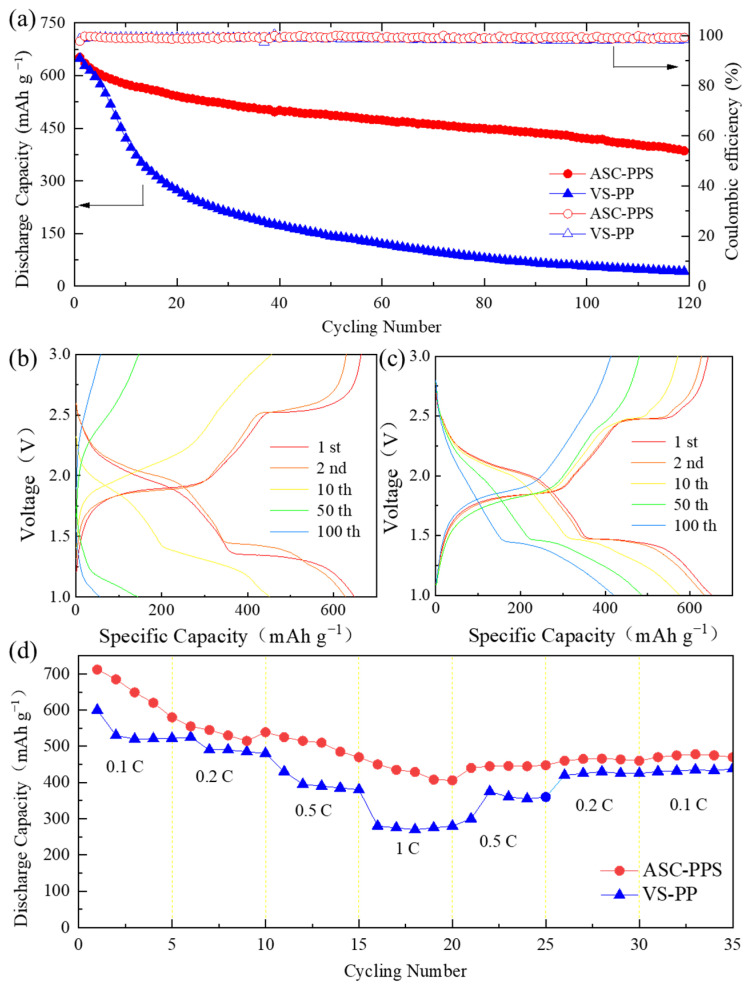
(**a**) Comparison of cycling performance at 0.5 C. Voltage–capacity curves of (**b**) VS-PP and (**c**) ASC-PPS. (**d**) Rate capability curves of VS-PP and ASC-PPS.

The synergistic design of cathode modification (ASC) and separator modification (PPS) significantly improved the cycling stability and rate performance of Li-FeS_2_ batteries. The dual-modified battery (ASC-PPS) exhibited an initial discharge capacity of 650 mAh g^−1^ at 0.5 C, with a capacity retention of 61.5% (400 mAh g^−1^) after 120 cycles, representing a 14% improvement over the control group (VS-PP, 47.5% retention, 190 mAh g^−1^) (Figure 2a). To contextualize these results within the broader field, our dual-modified Li-FeS_2_ battery (ASC-PPS) exhibits superior performance compared with recent reports, particularly in cycling stability and capacity retention under practical conditions; specifically, our battery’s 61.5% capacity retention after 120 cycles stands out against state-of-the-art reports. For FeS_2_ cathodes with chelating-type binders [40], 76.9% retention over 100 cycles at 0.2 C was reported; in contrast, our battery retains 61.5% over 120 cycles at a higher current density (0.5 C) without relying on specialized ether-based electrolytes or Li metal anodes, instead using a polymer matrix cathode and coated separator for stability. A Li-FeS_2_ battery with an electrospun MoS_2_-CNTs-PVA separator [49] retained 400 mAh g^−^^1^ after 200 cycles at 0.5 C. Our design delivers a higher initial capacity (650 vs. 587 mAh g^−^^1^) with comparable long-term stability, using a simpler doctor-blade coating process for separator modification, underscoring scalable performance advantages.

The impact of cathode materials on battery capacity density was substantial, as shown in Figure S1, where ASC significantly enhanced capacity retention compared with the unmodified cathode. In the control group (VS-PP, Figure 2b), the specific discharge capacity at the 2.5 V plateau decreased rapidly due to dual failure mechanisms: the PVDF binder’s lack of chemical anchoring causes sulfur loss, and low conductivity increases charge transfer resistance, and the unmodified PP separator enables polysulfide shuttling and lower ionic conductivity, exacerbating performance fade. In contrast, the full battery with the coated PPS separator (Figure 2c) exhibited two distinct discharge plateaus within the first 50 cycles at 0.5 C, indicating superior cycling stability. The voltage profile results indicated that the introduction of CNTs and the novel polyolefin binder significantly suppressed LiPS shuttling and improved the utilization of active materials, such as FeS_2_, during cycling.

Rate capability tests (Figure 2d) showed that the dual-modified battery retained 382 mAh g^−1^ at 1 C and rebounded to 426 mAh g^−1^ (69% of the initial capacity) when switched back to 0.1 C, whereas the control group only recovered to 45%. This confirms that the dual strategy effectively mitigated kinetic polarization.

**Figure 3:**
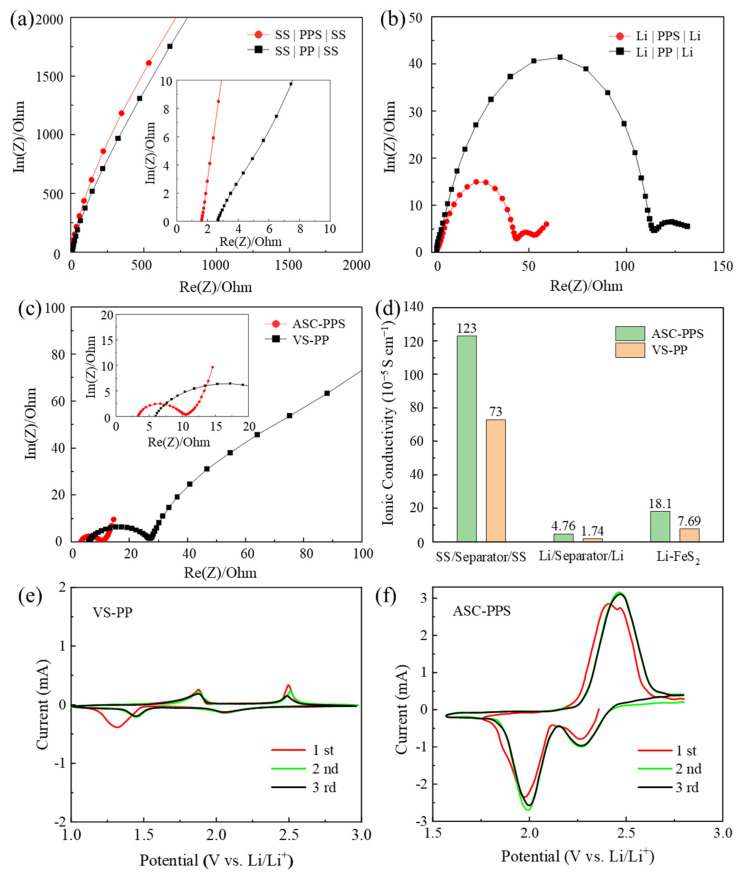
Nyquist plots of (**a**) SS|separator|SS, (**b**) Li|separator|Li, and (**c**) full cells; (**d**) comparison of ionic conductivities; CV curves of (**e**) VS-PP and (**f**) ASC-PPS.

Electrochemical impedance spectroscopy (EIS) was performed on three types of cells—steel symmetric cells (SS/separator/SS), lithium symmetric cells, and full cells—assembled with modified (ASC-PPS) and unmodified (VS-PP) components, with ionic conductivity measured at 25 °C. As shown in Figure 3a, the EIS results for the SS/separator/SS cells revealed that the internal resistances of the PPS and PP separators were 1.62 Ω and 2.74 Ω, respectively, with corresponding ionic conductivities of 1.23 × 10^−3^ S cm^−1^ and 7.3 × 10^−4^ S cm^−1^. The conductive carbon coating on PPS reduced charge transfer impedance and enhanced ionic conductivity without obstructing lithium-ion transport channels, thereby improving battery reversibility. Lithium symmetric cell tests (Figure 3b) showed that the internal resistances were 42 Ω (PPS) and 115 Ω (PP), with ionic conductivities of 4.76 × 10^−5^ S cm^−1^ and 1.74 × 10^−5^ S cm^−1^, respectively, indicating better interfacial compatibility between the coated separator and lithium metal due to improved ionic conductivity from the conductive carbon layer. For full cells (Figure 3c), the dual-modified system exhibited lower charge transfer resistance (11 Ω vs. 26 Ω) and higher ionic conductivity (1.81 × 10^−4^ S cm^−1^ vs. 7.69 × 10^−5^ S cm^−1^), confirming superior compatibility between the coated separator and ASC cathode. The results from the comparative plot of ionic conductivities among the three tested battery systems (Figure 3d) indicate that the PPS coating enhances ionic transport and reduces interfacial resistance, leading to higher reversible cycle capacity in Li-FeS_2_ batteries.

CV curves of the control (VS-PP) and dual-modified (ASC-PPS) groups are shown in Figure 3e,f. The VS-PP curve exhibited broadened peaks in the 1.5–2.5 V range, with a first-cycle peak separation (ΔEp) of ~170 mV and a peak current ratio (Ipa/Ipc) deviating from 1, indicating severe polarization and poor reversibility due to high interfacial resistance (Rct = 49 Ω in EIS) and side reactions from LiPS shuttling. In contrast, the ASC-PPS curve featured sharp and symmetric peaks, with ΔEp reduced to ~120 mV and overlapping peaks across multiple cycles, and Ipa/Ipc ≈ 0.92, approaching ideal reversibility. This improvement is attributed to the synergistic effects of the cathode’s three-dimensional conductive network (enhancing electron transport and structural stability) and the separator’s polysulfide adsorption (suppressing shuttling), which collectively optimized reaction kinetics, reduced interfacial polarization (ΔEp decreased by 30%), and improved reversibility.

### 3.2. Mechanism of Cathode Modification for Structural Degradation Suppression

As illustrated in Figure 1a, the ASC cathode incorporates PAN as a replacement for the conventional PVDF binder, forming a compact film morphology that establishes robust adhesion between various cathode components. This structural feature provides enhanced mechanical support, strengthens electrode adhesion, and effectively prevents active material detachment from the current collector during cycling, which is critical for mitigating the impact of volume variations. Concurrent with binder optimization, the integration of carbon nanotubes (CNTs) synergistically improves both the electrical conductivity and mechanical integrity of the electrode. CNTs construct a highly conductive network that interconnects FeS_2_ active particles, ensuring unimpeded electron transport throughout the electrode matrix. DC four-probe measurements confirm that the ASC cathode maintains superior electronic conductivity (8.476 S cm^−^^1^) during cycling, significantly exceeding the value of the VS cathode (1.251 S cm^−^^1^). Furthermore, CNTs form a three-dimensional (3D) network that enhances mechanical stability through multi-level stress buffering mechanisms: (1) macroscopic stress dispersion via a high-aspect-ratio (>10^3^) structural skeleton; (2) microscopic toughening enabled by hydrogen bonding between CNT hydroxyl groups and PAN nitrile moieties; and (3) elastic deformation capability (strain > 10%) to accommodate FeS_2_ volume expansion. This comprehensive reinforcement strategy minimizes structural degradation during cycling, thereby extending the cycle life of FeS_2_-based cathodes.

**Figure 4:**
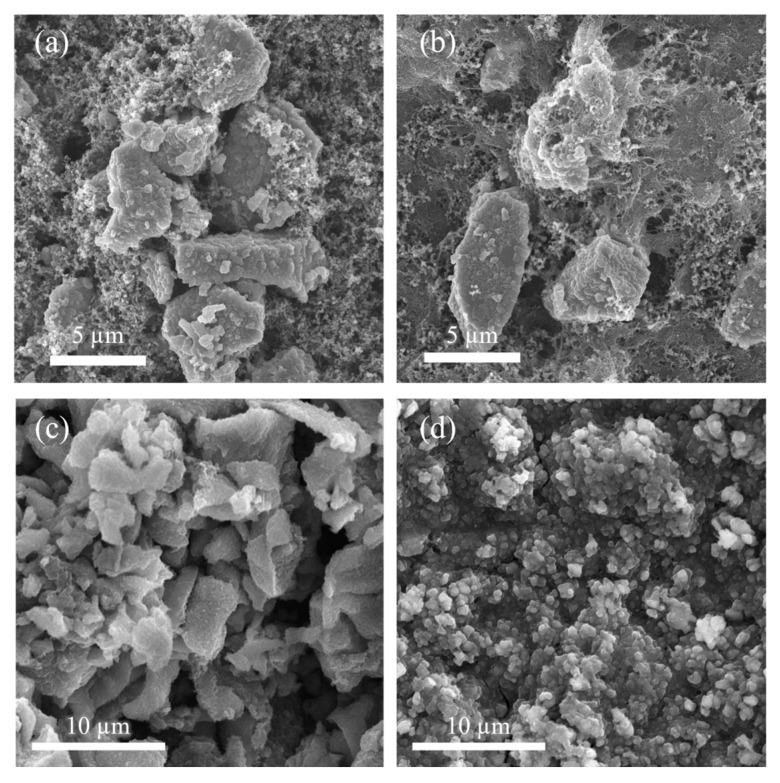
SEM morphology of the pristine (**a**) VS and (**b**) ASC cathodes and the disassembled cathodes from (**c**) VS-PP and (**d**) ASC-PPS batteries after 20 cycles.

Based on the SEM images of the ASC and VS cathode surfaces, it was observed that the components of the ASC cathode (Figure. 4b) were tightly interwoven by fibrous filaments due to the incorporation of PAN and CNTs. This highlights the crucial role of the PAN binder in providing strong adhesion between the current collector and active materials, as well as robust mechanical connections between particles. Simultaneously, the uniform dispersion of CNTs within the structure contributes to superior electronic conductivity. In contrast, the VS cathode (Figure 4a) exhibited insufficient particle interconnectivity.

Mechanical adhesion of the binder was quantified via 180° peel tests conducted at a peeling speed of 300 mm·min^−^^1^. The displacement–peel force curves of the peel test for the ASC (PAN binder) and VS (PVDF binder) cathodes are provided in Figure S3. The results revealed that the PAN binder exhibited an average peel force of 264.6 g·f^−^^1^, which is 73% higher than that of the PVDF binder (153.2 g·f^−^^1^). This enhanced adhesion arises from strong polar interactions between the nitrile groups (-C≡N) of PAN and the hydroxyl groups (–OH) on the aluminum current collector surface, reinforced by the “pinning effect” of the CNT network on PAN molecular chains. BET analysis further confirms the structural advantage: the ASC cathode has a higher specific surface area (9.2 m^2^ g^−^^1^) and smaller average pore size (41.4 nm) than the VS cathode (3.1 m^2^ g^−^^1^, 35.8 nm), enhancing electrolyte contact and buffering volume expansion. The superior adhesion prevents active material detachment during cycling, as evidenced by post-cycling SEM characterization: SEM imaging revealed that the control cathode (Figure 4c) had larger surface particles with dense cracks and particle detachment after cycling, indicating FeS_2_ fragmentation and isolated carbon domains. This was attributed to the lower adhesion strength of PVDF (153.2 g·f^−^^1^), which failed to withstand the mechanical stress from FeS_2_’s ≈160% volume expansion, leading to rapid sulfur dissolution from the cathode into the electrolyte during early cycles. These dissolved polysulfides hindered reactions and caused incomplete utilization of active materials, resulting in irregularly shaped, fragmented particles and electrode swelling with surface voids indicating active material loss. In contrast, the dual-modified cathode (Figure 4d) exhibited uniformly rounded particles with minimal agglomeration after cycling, with evenly distributed active materials and significantly improved structural integrity. This retention of morphology directly stems from the high adhesion strength of the PAN-CNT binder system (264.6 g·f^−^^1^), which anchored active materials firmly to the current collector. The uniform particle distribution confirmed that the combined introduction of CNTs and PAN effectively anchored active materials and sulfur on the electrode surface via chemical bonding and mechanical reinforcement, preventing solid sulfur from dissolving into the electrolyte to form polysulfides and reducing side reactions. Concurrently, the enhanced ionic and electronic conductivity of the cathode was confirmed by the high capacity retention and reversible cycling performance shown in Figure 2. The PAN binder stabilizes the cathode through dual interactions: (1) chemical anchoring between nitrile groups (-C≡N) and Fe^2+^ in FeS_2_ via coordinate bonding, which suppresses particle agglomeration and detachment [46], and (2) polar–polar interactions and S-π bonding between PAN’s polar groups and sulfur species (including pure sulfur), inhibiting sulfur dissolution and the shuttle effect, as supported by the strong polysulfide adsorption capacity of N-containing groups in PAN-based composites [17]. This synergistic effect explains the retained uniform particle distribution in post-cycling SEM images.

### 3.3. Suppression of Shuttle Effect via Separator Modification

**Figure 5:**
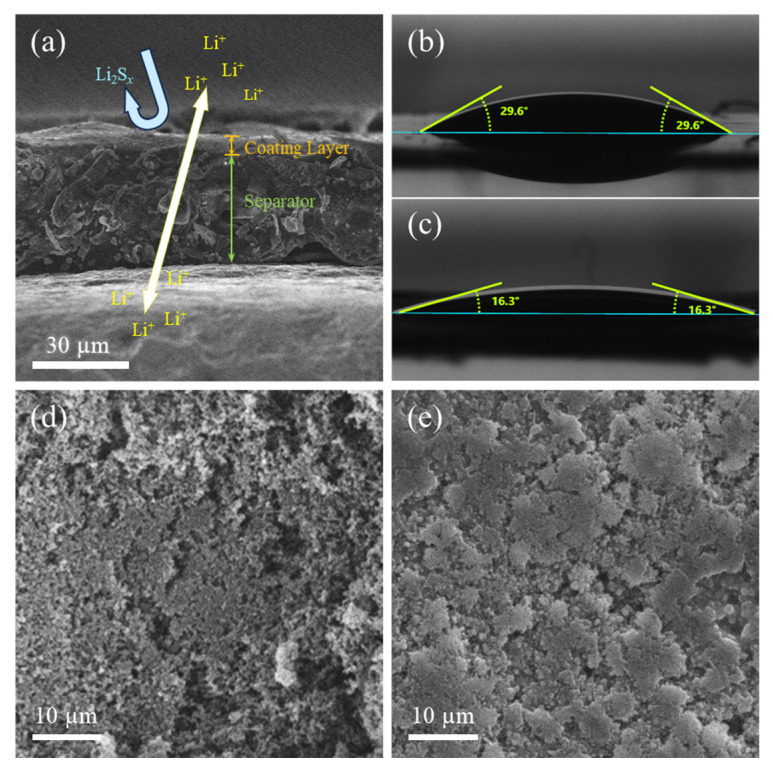
(**a**) Cross-sectional SEM of the PPS separator; contact angle measurements of (**b**) the untreated PP separator and (**c**) the modified PPS separator; SEM images of (**d**) the PP separator and (**e**) the PPS separator after 120 cycles.

The PPS separator’s coating (8 μm thickness, Figure 5a) exhibited a hydrophilic contact angle of 16.3° (Figure 5c), a 45% reduction from the unmodified separator (29.6°, Figure 5b), enhancing electrolyte wettability. We optimized the Super P content to 8 wt% to balance ionic conductivity and polysulfide retention: excessive carbon (>10 wt%) blocks ion channels (as observed in EIS with increased resistance), while insufficient carbon (<5 wt%) reduces adsorption capacity [41]. The initial surface morphology of PPS (Figure. 5d) showed a sparse porous network with a uniform coating, except for minor carbon agglomeration on the sides, providing critical structures for polysulfide adsorption. 

EDS mapping (Figure S2) and elemental content analysis (Table S1) of pristine and post-cycling separators provide quantitative insights into interfacial behavior: post-cycling PP separators exhibit substantial sulfur (3.49 at.%) and iron (0.9 at.%) migration, directly indicating unimpeded polysulfide shuttling and concomitant cathode degradation. In stark contrast, the PPS separator maintains stable fluorine content (consistent with PVDF coating integrity) while achieving significant sulfur enrichment (5.92 at.%)—1.7-fold higher than the PP control—and minimal iron migration (0.11 at.%). This marked difference in elemental distribution validates the PVDF-conductive carbon coating’s efficacy in polysulfide trapping and confirms enhanced cathode structural stability via suppressed active material loss. After 120 cycles at 0.5 C, the separator from the dual-modified full battery was disassembled, rinsed in DME, and dried, revealing a gel-like network structure on the cathode-facing surface (Figure 5e). EDS mapping (Figure S2) confirmed abundant sulfur species on the PPS surface, indicating that migrated polysulfides were trapped as irregular aggregates. The rough texture and porous gaps of these aggregates likely resulted from crystallization during polysulfide formation, potentially influencing electrochemical performance by limiting their dissolution and diffusion. 

The PVDF-coated separator enhances polysulfide adsorption primarily through dipole–dipole interactions: PVDF’s polar C-F bonds generate permanent dipoles, where fluorine’s δ^−^ charge electrostatically attracts the δ^+^ charge on sulfur in Li_2_S_x_ (4 ≤ x ≤ 8). Compared with alternative methods, dipole–dipole interactions outperform non-specific van der Waals-based trapping in porous carbon. This balance of selectivity and reversibility, combined with effective adsorption by the PPS coating via dipole interactions between polar groups and polysulfides, surpasses traditional separators relying solely on physical barriers. Integrating wettability optimization and chemical adsorption suppresses shuttling, boosting the Coulombic efficiency to 99.2% (Figure 2a) and contributing to the 61.5% capacity retention of the dual-modified battery.

## 4. Conclusions

This work presents a synergistic dual-strategy design to address the critical challenges of the polysulfide shuttle effect and cathode structural degradation in Li-FeS_2_ batteries, offering a scalable solution to advance their practical application.

To mitigate cathode structural degradation induced by 160% volume expansion and poor conductivity, a PAN-CNT composite cathode was developed. The PAN binder establishes strong chemical anchoring between its nitrile groups (-C≡N) and Fe^2+^ in FeS_2_, suppressing particle pulverization and sulfur dissolution. The 3D CNT network enhances electronic conductivity to 8.476 S cm^−^^1^ (≈7-fold higher than PVDF-based controls) and dissipates expansion stress, with BET analysis confirming a higher surface area (9.2 m^2^ g^−^^1^) and optimized pore size (41.4 nm) that enhance electrolyte contact and volume buffering. This design achieves 73% higher adhesion strength (264.6 g·f^−^^1^) than PVDF, preventing active material detachment.

A polyvinylidene fluoride (PVDF)-conductive carbon-coated separator (PPS) was designed to suppress the polysulfide shuttle effect. PVDF’s polar C-F groups trap polysulfides through dipole–dipole interactions, with Super P providing electron pathways for reutilizing trapped species. This separator achieves high ionic conductivity (1.23 × 10^−^^3^ S cm^−^^1^) and hydrophilicity (16.3° contact angle) to facilitate unobstructed Li^+^ transport, with EDS mapping verifying post-cycling sulfur enrichment and minimal Fe migration (0.11 at.%), confirming effective polysulfide adsorption and cathode stability.

The electrochemical results validate the synergistic efficacy: the modified battery delivers an initial discharge capacity of 650 mAh g^−^^1^ at 0.5 C, retaining 61.5% after 120 cycles (14% higher than controls) with 99.2% Coulombic efficiency and reduced charge transfer resistance. Microstructural characterizations confirm minimal post-cycling cracks, directly linking structural preservation to performance improvements. This polymer-based design synergistically overcomes Li-FeS_2_ bottlenecks, offering a low-cost, scalable pathway to enhance cycling stability and energy density for advancing conversion-type battery commercialization.

## Data Availability

The original contributions presented in this study are included in the article/Supplementary Materials. Further inquiries can be directed to the corresponding author(s).

## Supplementary Materials

The following supporting information can be downloaded at https://www.mdpi.com/article/10.3390/ma18174058/s1. Figure S1: Comparison of cycling performance at 0.5 C between the modified and unmodified cathodes. Figure S2: EDS scanning results of sulfur element on the surface of the separator after disassembly. Table S1: Comparison of atomic content (at.%) for each element before and after cycling. Figure S3: The displacement-peel force curves of the peel test for ASC and VS cathode sheets.
