# Supplementary material for: Dual-Strategy Design Based on Polymer–Matrix Composite Cathode and Coated Separator for High-Performance Lithium–Iron Disulfide Batteries
Source: Materials (Basel). 2025 Aug 29;18(17):4058. doi: 10.3390/ma18174058 (PMC12429738; doi:10.3390/ma18174058)
Supplement: Supplementary file 1 [file materials-18-04058-s001.zip › materials-3819922-supplementary.pdf]

materials

Supporting Information

Dual-Strategy Design Based on Polymer Matrix Composite Cathode and Coated Separator for High-Performance Lithium–Iron Disulfide Batteries

Fan Zhang [1], Qiang Lu [2], Jiachen Li [1,3], Qiongyue Zhang [1], Haotian Yu [1], Yahao Wang [1], Jinrui Li [1], Haodong Ren [1], Huirong Liang [1], Fei Shen [2,*] and Xiaogang Han [2,*]

[1] School of Electric Power, Civil Engineering and Architecture, Shanxi University, Taiyuan 030000, China; fanzhang@sxu.edu.cn (F.Z.); 15343481638@163.com (J.L.); 202424314047@email.sxu.edu.cn (Q.Z.); 202223503038@email.sxu.edu.cn (H.Y.); 202323504037@email.sxu.edu.cn (Y.W); lijinrui1@sxu.edu.cn (J.L.); renhaodong1@sxu.edu.cn (H.R.); lianghui3@sxu.edu.cn (H.L.)
[2] State Key Laboratory of Electrical Insulation and Power Equipment, School of Electrical Engineering, Xi'an Jiaotong University, Xi'an 710049, China; 3123304252@stu.xjtu.edu.cn
[3] Shanxi Yudean Energy Company Limited, Taiyuan 030006, China.
* Correspondence: feishen@xjtu.edu.cn (F.S.); xiaogang.han@xjtu.edu.cn(X.H.)

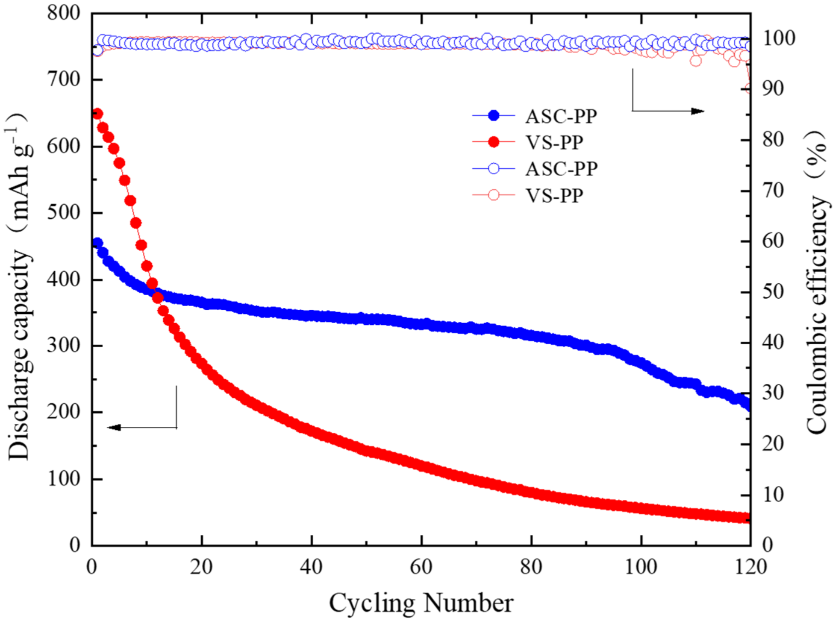

Figure S1. Comparison of cycling performance at 0.5 C between the modified and unmodified cathodes.

 https://doi.org/10.3390/xxxxx

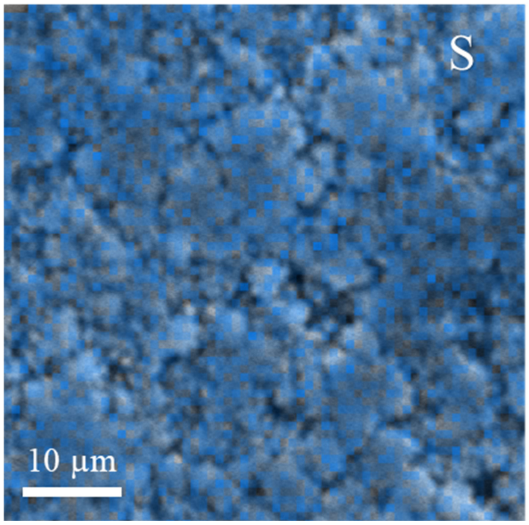

Figure S2. EDS scanning results of sulfur element on the surface of the separator after disassembly.

Table S1. Comparison of atomic content (at.%) for each element before and after cycling.

Elements	PP separator		PP separator	
	Before cycling	After disassembly	Before cycling	After disassembly
C	99.78	94.55	88.11	82.31
F	--	--	11.14	10.55
O	0.21	1.06	0.55	1.20
S	--	3.49	0.14	5.92
Fe	--	0.90	0.06	0.11

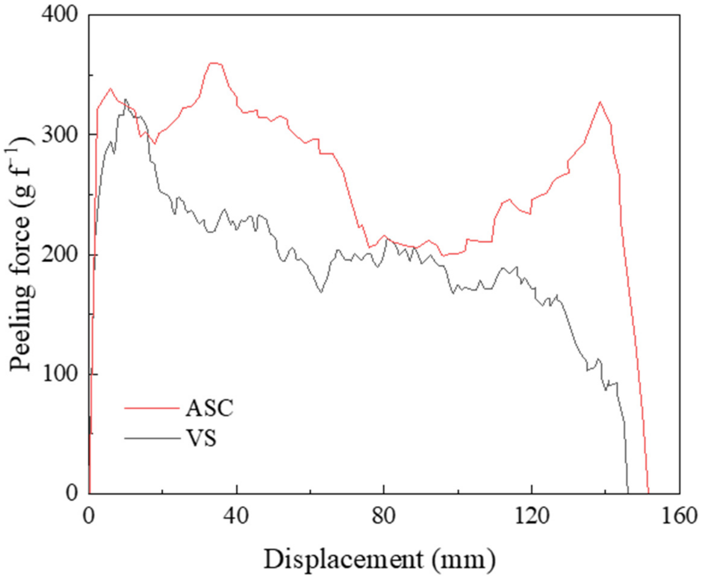

Figure S3. The displacement–peel force curves of the peel test for ASC and VS cathode sheets.
